# Detecting clinical cases of binge eating in diabetes care: Introducing the Diabetes Eating Problem Survey‐10 (DEPS‐10) for type 1 and type 2 diabetes

**DOI:** 10.1111/dme.70060

**Published:** 2025-05-29

**Authors:** Laura Yvonne Klinker, Andreas Schmitt, Dominic Ehrmann, Bernhard Kulzer, Norbert Hermanns

**Affiliations:** ^1^ Research Institute Diabetes Academy Mergentheim (FIDAM) Bad Mergentheim Germany; ^2^ Diabetes Center Mergentheim (DZM) Bad Mergentheim Germany; ^3^ German Center for Diabetes Research (DZD) München‐Neuherberg Germany; ^4^ Department of Clinical Psychology and Psychotherapy University of Bamberg Bamberg Germany

**Keywords:** assessment tool, binge eating disorder, diabetes mellitus, disordered eating behaviour, questionnaire, screening

## Abstract

**Aims:**

Binge eating disorders (BED) are underdiagnosed in diabetes care, despite being the most common eating problem for diabetes patients. While diabetes‐specific screening for disordered eating behaviour is recommended, the only diabetes‐specific instrument available, Diabetes Eating Problem Survey‐Revised (DEPS‐R), focuses on type 1 diabetes and rapid‐acting insulin, limiting its use across diabetes types and treatment regimens. This study aimed to develop a non‐insulin version of the DEPS‐R and evaluate its screening performance for BED in people with type 1 and type 2 diabetes.

**Methods:**

The DEPS‐R was reduced to 10 non‐insulin‐specific items (DEPS‐10). As part of the ongoing pro‐mental study, 679 people with type 1 or type 2 diabetes completed the baseline survey and took part in diagnostic interviews to assess BED. The screening performance of the DEPS‐10 was tested via receiver operating characteristic (ROC) curve analysis and compared with DEPS‐R and food‐related items of the Problem Areas In Diabetes (PAID).

**Results:**

*N* = 24 participants (total = 3.5%; type 1 = 2.9%, type 2 = 4.3%) were diagnosed with a current BED. The DEPS‐10 performed well in screening for BED (area under the curve [AUC] = 0.92, *p* < 0.001) comparable with the DEPS‐R (AUC = 0.92, *p* < 0.001) and exceeded the performance of food‐related PAID items (AUC = 0.82, *p* < 0.001). A cut‐off score of ≥15 showed optimal sensitivity and specificity in BED screening. People who met the cut‐off had significantly higher BMI and HbA1c and more diabetes distress, depressive and anxiety symptoms.

**Conclusions:**

DEPS‐10 is a reliable screening instrument for BED. Its associations with glycaemic and mental health outcomes reflect its good construct validity comparable to DEPS‐R.

**What's New?**

- Diabetes‐specific instruments are needed to reliably assess disordered eating in people with diabetes.
- The diabetes‐specific scale DEPS‐R only focuses on type 1 diabetes and rapid‐acting insulin, limiting its use among people with type 2 diabetes and preventing comparisons across diabetes type groups.
- This study introduces a revised, non‐insulin‐specific version of the DEPS‐R, suitable for screening for binge eating and dysfunctional eating behaviour in people with diabetes of both major types. The revised 10‐item scale showed excellent screening properties for BED.
- A cut‐off score ≥ 15 was identified as a suitable threshold for screening of binge eating behaviour in people with diabetes.

## 1 INTRODUCTION

Binge eating (BE) is the most common dysfunctional eating behaviour among people with type 1 and type 2 diabetes.^1^ According to the Diagnostic and Statistical Manual of Mental Disorders‐5 (DSM‐5), BED is defined by eating unusually large amounts of food in a discrete period of time while experiencing a loss of control at least once a week for at least 3 months.^2^ To qualify as a binge eating episode, at least three of the following characteristics should be present: (1) eating faster than normal; (2) eating until feeling uncomfortably full; (3) eating large amounts of food without physical hunger; (4) eating alone because of feeling embarrassed; and (5) feeling disgusted, depressed or guilty after overeating. An important criterion for binge eating disorders (BED) is the absence of compensatory behaviours like purging or insulin omission. Point prevalence rates for BED have ranged from 1.2% to 12.2% in type 2 diabetes across studies.^3^, ^4^, ^5^, ^6^, ^7^ For people with type 1 diabetes, the available evidence is mainly based on studies of adolescents, reporting prevalence rates ranging from 7.9%^8^ to 17.7%.^9^

BE has been associated with elevated HbA1c levels^10^ and increased post‐prandial glucose excursions.^11^ In a study by Huisman et al., HbA1c among people with diabetes and BE was 63 ± 12 mmol/L (7.9% ± 3.3%) compared with 57 ± 11 mmol/L (7.4% ± 3.2%) in people with diabetes without BE. Aside from the significant negative impact of BE on HbA1c levels, Moscovich et al. observed an increased negative change in affect after binge episodes compared with non‐binge episodes.^11^ Mental health problems, such as depression,^12^ elevated diabetes distress, and anxiety symptoms, are common comorbidities for people with BE.^1^, ^8^, ^13^ De Zwaan reviewed studies comparing obese individuals with and without BE and reported that among those with BE, 33%–91% had experienced an affective disorder and 9%–46% an anxiety disorder in their lifetime^12^ compared with 8%–57% and 1%–26%, respectively, in obese individuals without BE.

Despite the glycaemic and co‐morbid mental health problems, BED and BE remain underdiagnosed in people with diabetes.^4^, ^6^, ^14^ Significant healthcare professional bias towards BE and people with BE has been reported, which could negatively affect screening and detection of BE.^4^ To address these challenges, the American Diabetes Association recommends screening for disordered eating in people with diabetes along with screening for other mental health concerns, such as diabetes distress, depression, and anxiety.^15^ Screening with generic questionnaires is not advised for people with diabetes, because they may lead to under‐ and overreporting of disordered eating, neglecting diabetes‐specific challenges in eating behaviour.^16^ General scales for disordered eating (e.g., the Eating Disorder Inventory‐2^17^) do not account for diabetes‐specific behaviours. Specific BE questionnaires, for example the Binge Eating Scale (BES),^18^ may overestimate binge eating in individuals with diabetes due to their experienced dietary restraints. Therefore, the use of a diabetes‐specific instrument, such as the Diabetes Eating Problem Survey‐Revised (DEPS‐R), is recommended.^13^, ^19^, ^20^

The DEPS‐R was developed for use among individuals with type 1 diabetes; thus, six of the 16 items are specific to intensified insulin therapy.^19^ The DEPS‐R is only suitable for people with rapid‐acting insulin treatment, which has led to a lack of diabetes‐specific screening for many people with type 2 diabetes. To the authors' knowledge, only a few studies have tested the screening qualities of the DEPS‐R for eating disorders via the gold standard of clinical diagnostic interviews.^21^, ^22^ Ryman et al. reported low specificity (25%) of the DEPS‐R for clinically assessed eating disorders meeting DSM‐IV criteria.^21^ The limitations of the DEPS‐R in specificity and the lack of diabetes‐specific screening for people without intensified insulin therapy may contribute to the low detection rate of BED and BE in clinical care.

To facilitate the assessment and detection of BE and BED, and support personalised diabetes care, a reliable and valid assessment scale to measure eating problems is needed that is suitable for both type 1 and type 2 diabetes with or without intensified insulin treatment. In this study, we aim to close this gap by introducing a revised version of the DEPS‐R: DEPS‐10. DEPS‐10 is a shortened version of the DEPS‐R without insulin‐specific items, allowing screening of disordered eating regardless of diabetes type and treatment regimen. In the present study, its screening performance for BED in people with diabetes was evaluated. Unlike BED screening tools, the DEPS‐10 integrates diabetes‐specific behaviours (e.g., avoiding checking glucose levels) that may not be captured by generic instruments. Although the present study focuses on BED, the DEPS‐10 is designed to have broader relevance: maintaining the DEPS‐R's conceptual scope, items addressing non‐insulin‐specific compensatory behaviours were preserved, enabling inclusive screening of disordered eating beyond BED. We tested the screening qualities of the instrument for BED via clinical diagnostic interviews among a large sample of people with type 1 and type 2 diabetes. In addition, we compared the screening performance of the revised DEPS‐10 with that of the former DEPS‐R and with that of the items on food‐related problems of the Problem Areas in Diabetes Scale (PAID), a well‐established screening tool for diabetes distress.^23^, ^24^

To address timely challenges in routine care and increase specificity, a stepwise approach using the food‐related items of the PAID as a primary screening tool, followed by the revised DEPS‐10, was tested and evaluated.

## 2 METHODS

The analysed data were collected as part of the pro‐mental study, a prospective observational study conducted in secondary and tertiary health care centres in Germany. The study assesses the course of person‐reported outcomes (PRO) among people with type 1 and type 2 diabetes and their associations with parameters of glycaemic control. A key element of the study is clinical diagnostic interviews conducted at baseline. The study was approved by the ethics committee of the German Psychological Association (HermannsNorbert: 2022‐07‐14VADM). The current analysis uses data collected from May 2023 to July 2024.

### 2.1 Procedures

Participants were approached via their healthcare professionals and informed about the pro‐mental study by the research team. Inclusion criteria were informed consent, type 1 or type 2 diabetes, diabetes duration ≥1 year, aged 18–80 years, and sufficient German language skills. Exclusion criteria were relevant impaired cognitive functioning, serious illness confounding the results, being bedridden, and missing informed consent.

Structured clinical diagnostic interviews were conducted to assess mental disorders, particularly anxiety, affective, and eating disorders.^25^ The interviews were conducted via phone call or in person during a hospital stay using the Mini‐DIPS Open Access.^25^ BED was diagnosed based on the DSM‐5 criteria (Table S1).

The Mini‐DIPS Open Access refers to the diagnostic criteria of the ICD‐10 and DSM‐5 in its items and assesses point, 12‐month, and lifetime prevalence. The interviews were conducted by psychologists and trained psychological assistants.

### 2.2 Diabetes Eating Problem Survey‐Revised (DEPS‐R)

The DEPS‐R consists of 16 items assessing diabetes‐specific eating behaviours on a 6‐point Likert scale ranging from 0 (‘never’) to 5 (‘always’). The DEPS‐R shows good psychometric properties in adults, with a Cronbach's alpha of 0.84, and it correlates significantly with the Eating Disorder Examination‐Questionnaire (EDE‐Q), indicating good construct validity.^26^ A cut‐off score of ≥20 was reported for significant disordered eating.^19^ Six items address insulin purging attitudes and behaviours only applicable for people with rapid‐acting insulin treatment (Table S2). For participants without such treatment, the DEPS‐R was reduced to a 10‐item version (DEPS‐10) covering loss of control in eating, dietary and purging behaviour, and difficulties in diabetes management.

### 2.3 Problem Areas in Diabetes (PAID)

The 20‐item PAID addresses four different problem areas that are ranked on a 5‐point Likert scale ranging from 0 (‘not a problem’) to 4 (‘serious problem’).^24^, ^27^ A score of ≥40 serves as a cut‐off for elevated diabetes distress. Three scale items (Items 4, 5, and 11) address food‐related problems^27^; ‘feelings of deprivation regarding food’, ‘feeling constantly concerned about food and eating’, as well as ‘uncomfortable social situations’ with ‘people telling you what to eat’.

Healthcare records were consulted, including the HbA1c. The online survey comprised demographic data, validated questionnaires assessing diabetes distress (PAID), depressive (Patient Health Questionnaire, PHQ‐9), and anxiety symptoms (General Anxiety Scale, GAD‐7), and eating behaviour (DEPS‐R and DEPS‐10).

### 2.4 Statistical Analysis

A receiver operating characteristic (ROC) curve analysis evaluated the screening performance of the DEPS‐10 for BED point prevalence, and identified an optimal cut‐off using the Youden Index.^28^ The screening performance of the DEPS‐10 was compared with that of the DEPS‐R among participants with rapid‐acting insulin treatment, and with that of the PAID food‐related items in the total sample. Specificity, sensitivity, and positive and negative predictive values (PPV and NPV) were calculated for all measures. A stepwise screening approach, using the food‐related PAID items as a primary screener followed by the DEPS‐10, was also evaluated via the previously mentioned metrics.

A stepwise binomial logistic regression analysis was performed to predict the point prevalence of BED via DEPS‐10, controlling for demographics (sex, age, type of diabetes, HbA1c, BMI and diabetes complications: first block) and food‐related items of the PAID (second block). DEPS‐10 and PAID scores were *z*‐standardised to enable comparisons.

Psychometric properties of the DEPS‐10, including construct validity (via Spearman correlations), exploratory factor analysis, item‐total correlations and internal consistency (Cronbach's alpha), were evaluated (Tables S4–S8).

## 3 RESULTS

### 3.1 Sample

**FIGURE 1:**
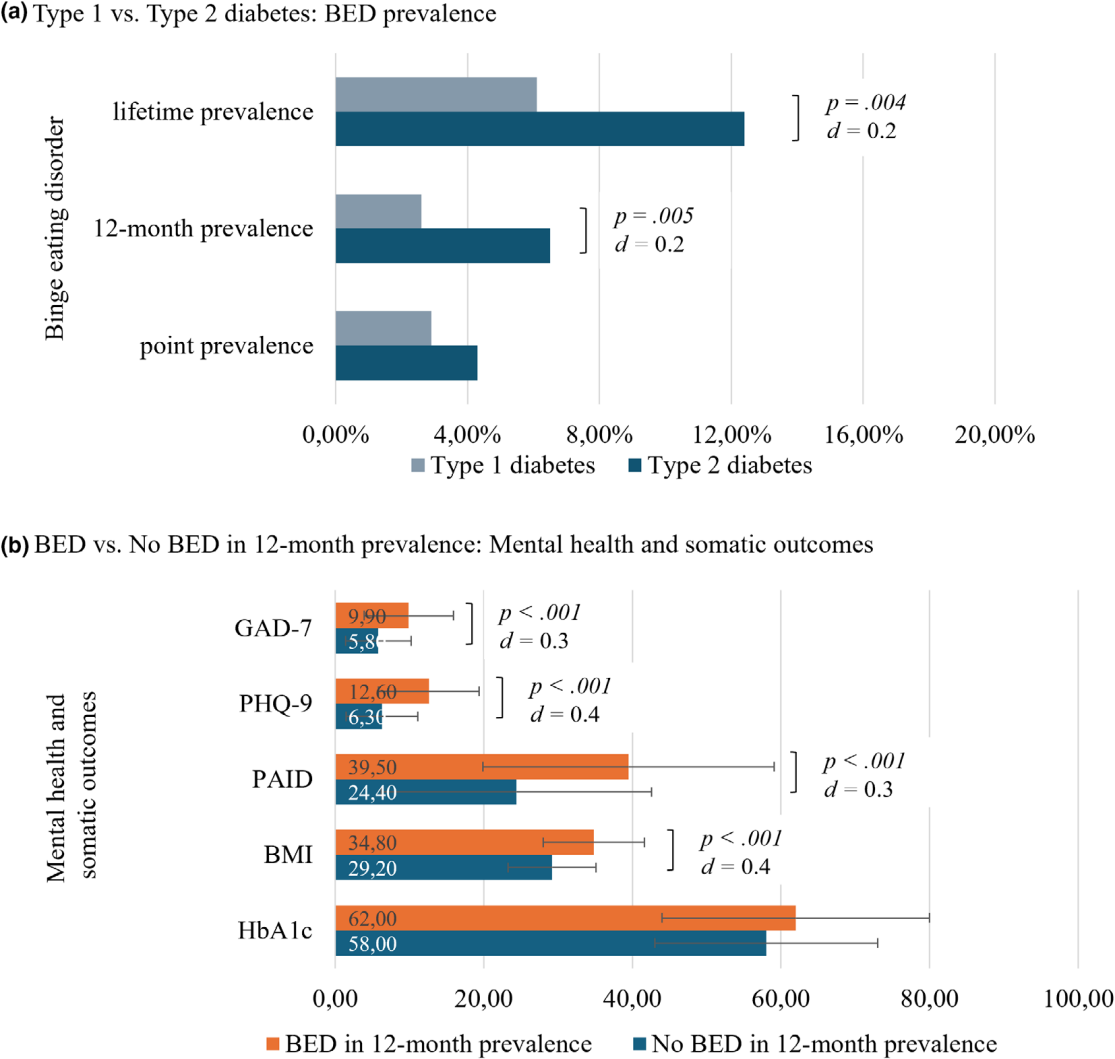
BED prevalence rates and psychosomatic outcomes. BED, binge eating disorder; BMI, body mass index; error bars, standard deviation; GAD‐7, General Anxiety Disorder‐7; HbA1c, haemoglobin A1c (mmol/mol); PAID, Problem Areas In Diabetes; PHQ‐9, Patient Health Questionnaire‐9. Mann–Whitney *U*‐tests were conducted. Figure 1a depicts prevalence in percentage, Figure 1b mean scores with standard deviation. More detailed information can be found in Table S3.

*N* = 679 participants were included in this analysis. Descriptive data can be found in Table 1. Twenty‐four participants (3.5%) met all criteria for BED at the time of the interview, and 4.4% (30 participants) had met the criteria for BED in the preceding 12 months. Participants with type 2 diabetes had higher prevalence rates of BED than participants with type 1 diabetes (Figure 1a; Table 1).

**TABLE 1:** Sample characteristics and prevalence of disordered eating.

	All (*n* = 679)	Type 1 diabetes (*n* = 345) 50.8%	Type 2 diabetes (*n* = 322) 47.4%
Age (years)	53.8 ± 16.1	46.1 ± 16.3	61.9 ± 11.2
Women (%)	330 (48.6%)	182 (52.8%)	142 (44.1%)
Men (%)	349 (51.4%)	163 (47.2%)	180 (55.9%)
Insulin treatment			
No insulin treatment	128 (18.9%)	345 (100%)	125 (38.8%)
Insulin treatment	551 (81.1%)		197 (61.2%)
Type 3 diabetes^a^	12 (1.8%)		
GLP‐1 therapy	137 (20.2%)	2 (0.6%)	134 (41.6%)
Diabetes duration (years)	18.7 ± 2.2	21.9 ± 13.4	15.3 ± 9.5
HbA1c (mmol/mol)	58 ± 15	59 ± 17	56 ± 14
(%)	(7.4 ± 1.4)	(7.6 ± 1.5)	(7.3 ± 1.3)
BMI	29.5 ± 6.1	27.3 ± 5.1	31.9 ± 6.2
Obesity (BMI ≥30)	263 (38.7%)	85 (24.6%)	176 (54.7%)
PAID			
Sum score	25.1 ± 18.5	26.8 ± 19.3	23.0 ± 17.2
Food‐related problems (max. = 12)	3.4 ± 2.8	3.2 ± 2.7	3.6 ± 2.8

Disordered eating				*p*	*d*
BED					
Point prevalence	24 (3.5%)	10 (2.9%)	14 (4.3%)	*0.182*	0.1
12‐month prevalence	30 (4.4%)	9 (2.6%)	21 (6.5%)	*0.005*	0.2
Lifetime prevalence	61 (9.0%)	21 (6.1%)	40 (12.4%)	*0.004*	0.2
*BE*					
Point prevalence	33 (4.9%)	12 (3.5%)	21 (6.5%)	*0.108*	0.1
12‐month prevalence	44 (6.5%)	12 (3.5%)	32 (9.9%)	*0.001*	0.3
Lifetime prevalence	82 (12.1%)	31 (9.0%)	50 (15.5%)	*0.010*	0.2
DEPS‐10	8.8 ± 6.5	8.4 ± 6.4	10.0 ± 6.1	*0.002*	6.2
DEPS‐R (*n* = 458^a^)	12.0 ± 9.7	11.7 ± 9.6	13.4 ± 9.8	*0.076*	9.6
*DEPS‐R* ≥ 20					
(*n* = 458^b^)	54 (11.8%)	40 (11.6%)	14 (11.9%)	*0.809*	0.02

*Note*: Values are means (M) ± standard deviation (SD), numbers in parentheses are column percentages. *χ* ^2^‐tests were conducted for all dichotomous variables and *t*‐tests for interval‐scaled variables (DEPS‐10 and DEPS‐R).

Abbreviations: BE, binge eating behaviour; BED, binge eating disorder; BMI, body mass index; *d*, effectsize Cohen's d; DEPS‐10, Diabetes Eating Problem Survey‐10; DEPS‐R, Diabetes Eating Problem Survey‐Revised; GLP‐1 therapy, glucagon‐like peptide‐1 therapy; HbA1c, haemoglobin A1c; PAID, Problems Areas in Diabetes Scale; *p*, *p*‐value.

^a^ Data of participants with type 3 diabetes (2%) were included in the study but did not influence the findings.

^b^ Only participants with insulin‐dependent diabetes answered the DEPS‐R.

The prevalence of dysthymia or a depressive episode in the last 12 months was three times higher for people with BED than for people without BED in 12‐month prevalence (36.7% vs. 10.3%, *p* < 0.001, *d* = 0.3). BED in 12‐month prevalence was associated with a significantly higher BMI (34.8 ± 6.8 vs. 29.2 ± 5.9, *p* < 0.001, *d* = 0.4) and more depressive (12.6 ± 6.8 vs. 6.3 ± 4.8, *p* < 0.001, *d* = 0.4) and anxiety symptoms (9.9 ± 6.0 vs. 5.8 ± 4.4, *p* < 0.001, *d* = 0.3; Figure 1b). HbA1c was higher for people with BED, but the difference was not significant (62 ± 18 mmol/mol vs. 58 ± 15 mmol/mol, i.e. 7.8 ± 3.8% vs. 7.5 ± 3.5%, *p* = 0.13, d = 0.1), which may be due to a median time gap of 27 days (IQR: 69 days) between clinical interview and healthcare records.

### 3.2 ROC curve analysis

**FIGURE 2:**
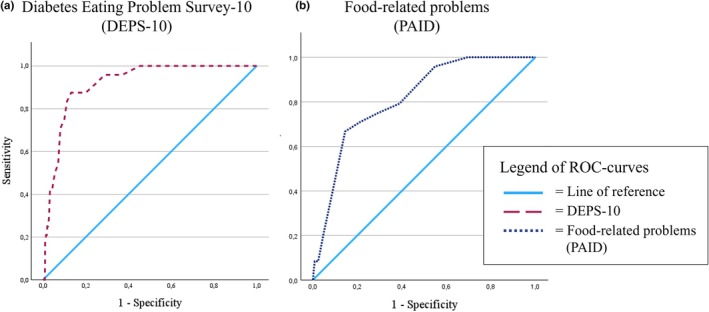
ROC curves of the DEPS‐10 and food‐related items of the PAID in predicting the point prevalence of BED. BED, binge eating disorder; PAID, Problem Areas In Diabetes Scale; ROC, receiver operating classification.

DEPS‐10 showed high internal consistency (Cronbach's *α* = 0.852). Further psychometric characteristics are available in Tables (S4–S8). The DEPS‐10 showed a good screening performance with AUC = 0.92 (CI: 0.88; 0.96) (Figure 2) and an optimal cut‐off value of 15. In comparison, the food‐related PAID items had an AUC = 0.82 (CI: 0.74; 0.89), and a cut‐off score ≥ 6 was identified (Table 2). With a PPV of 19.6%, five clinical interviews are needed after DEPS‐10 screening to identify one BED case. The PPV was higher than for screening via PAID items (14.5%), requiring seven interviews to identify one BED case. In the subsample of people with rapid‐acting insulin treatment, DEPS‐10 screening would require six clinical interviews to diagnose one BED case, compared with eight diagnostic interviews after DEPS‐R‐screening (PPV: 16.4% vs. 13.0%). In this subsample, the DEPS‐10 showed higher sensitivity than the DEPS‐R (91% vs. 64%) and comparable specificity (89%).

**TABLE 2:** AUC, sensitivity, specificity, and predictive values (PPV, NPV) of DEPS‐10 and PAID.

Scales	AUC (95% CI)	*p*	Sensitivity	Specificity	PPV	NPV	Optimal cut‐off score
DEPS‐10 (all)	0.92 (0.88, 0.96)	*<0.001*	87.5%	86.9%	19.6%	99.5%	≥15
Food‐related Problems (PAID)	0.82 (0.74, 0.89)	*<0.001*	66.7%	85.6%	14.5%	98.6%	≥6
DEPS‐10 (ICT)	0.93 (0.86, 0.99)	*<0.001*	90.9%	88.6%	16.4%	99.7%	≥15
DEPS‐10 (no ICT)	0.91(0.85, 0.96)	*<0.001*	84.6%	83.2%	23.9%	98.9%	≥ 15
DEPS‐R	0.92 (0.86, 0.97)	*<0.001*	63.6%	89.4%	13.0%	99.0%	≥20

Abbreviations: AUC, area under the curve; PPV, positive predictive value; NPV, negative predictive value; *p*, *p*‐value; PAID, Problem Areas In Diabetes Scale; DEPS‐10, Diabetes Eating Problem Survey‐10; ICT, intensified conventional insulin therapy; DEPS‐R, Diabetes Eating Problem Survey‐Revised.

**FIGURE 3:**
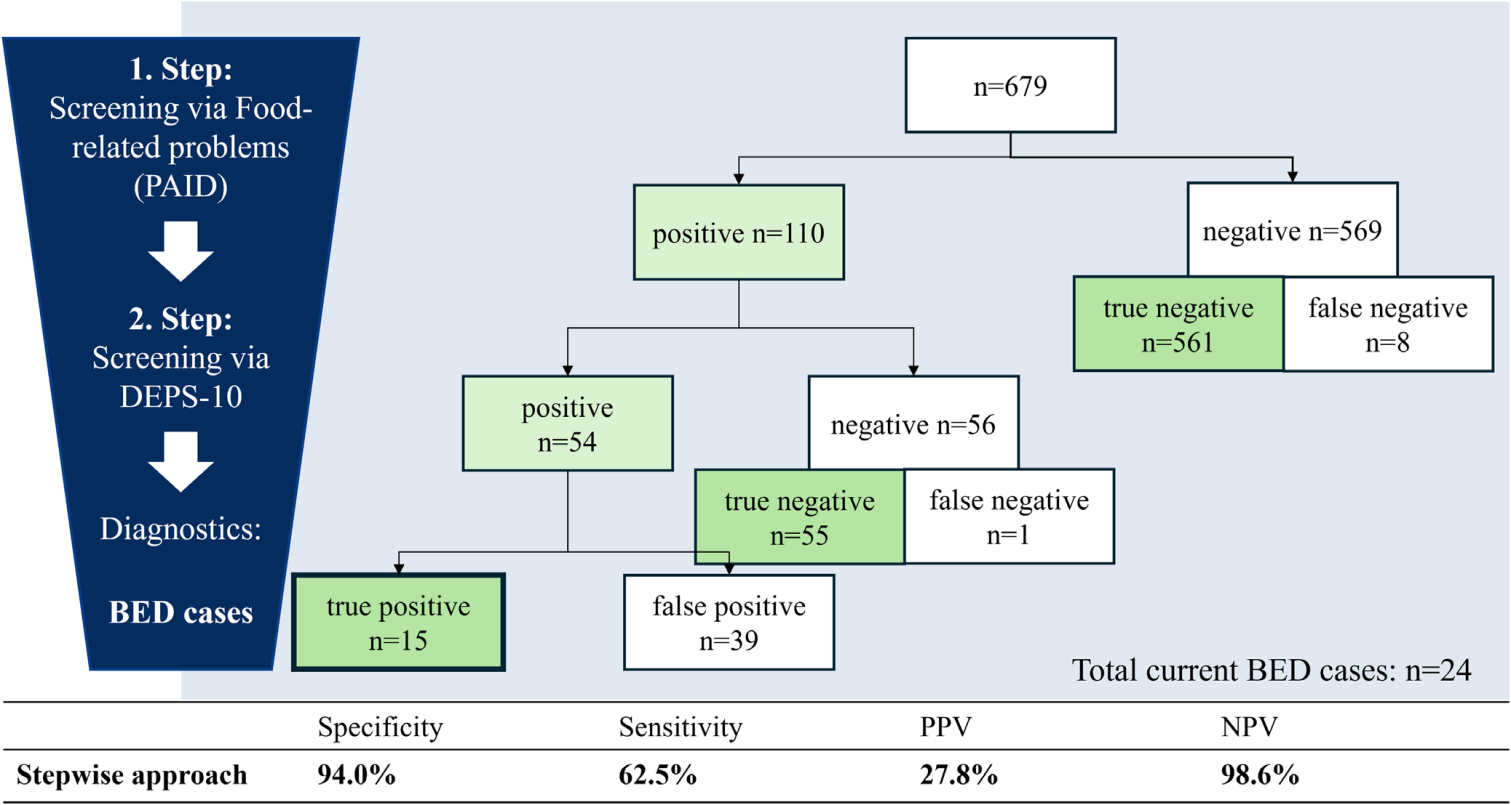
Stepwise approach using food‐related PAID items as primary and DEPS‐10 as secondary screening instrument. BED, binge eating disorder; DEPS‐10, Diabetes Eating Problem Survey‐10; NPV, negative predictive value; PAID, Problem Areas In Diabetes Scale; PPV, positive predictive value.

In all, 15.2% of the total sample reached the cut‐off ≥ 15 for the DEPS‐10, including 12.1% of participants with type 1 diabetes and 18.9% of participants with type 2 diabetes. Participants receiving GLP‐1 therapy showed higher DEPS‐10 scores (20.4% DEPS‐10 ≥ 15) than participants without GLP‐1 therapy (*M* ± *SD* = 10.9 ± 5.7 vs. 8.7 ± 6.4, *p* < 0.001). Participants with DEPS‐10 ≥ 15 had significantly higher HbA1c and BMI, as well as a significantly higher psychological burden with more depressive and anxiety symptoms and diabetes distress, than those with DEPS‐10 ≤ 15 (Figure 3). Of participants with DEPS‐10 ≥ 15, 22% had also had a depressive episode or dysthymia in the preceding 12 months (Table S8).

### 3.3 Stepwise approach: Food‐related problems (PAID) and DEPS‐10

**FIGURE 4:**
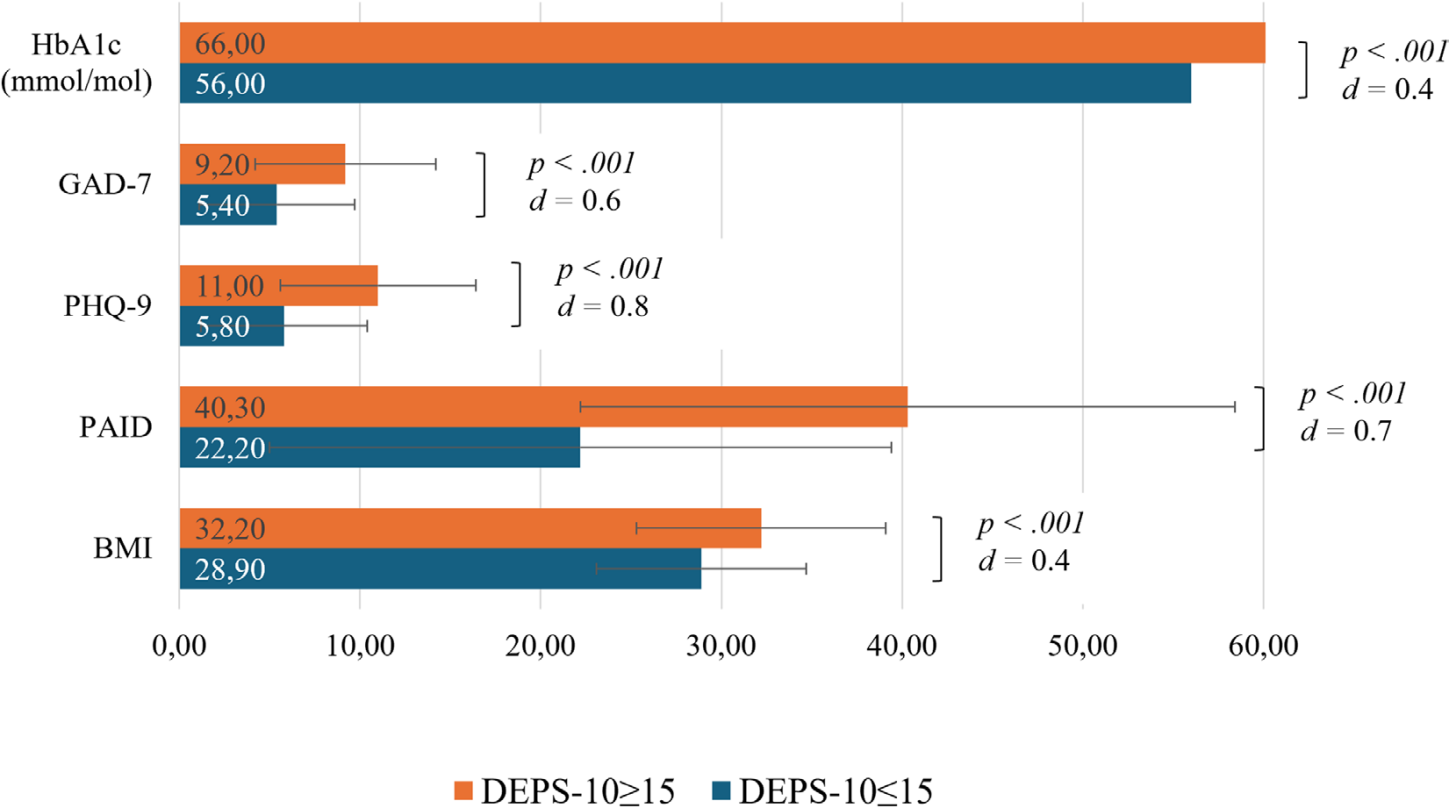
DEPS‐10 ≥ 15 vs DEPS‐10 ≤ 15: Glycaemic and mental health outcomes. BMI, body mass index; DEPS‐10, Diabetes Eating Problem Survey‐10; error bars, standard deviation; GAD‐7, General Anxiety Disorder‐7; HbA1c, haemoglobin A1c; PAID, Problem Areas In Diabetes Scale; PHQ‐9, Patient Health Questionnaire‐9.

In this sample, screening with PAID food‐related items identified 110 BED cases, including 16 true cases and 94 false positives. Adding the DEPS‐10 screening reduced the total number of cases identified to 54, of which 15 participants met full BED criteria in the clinical interview (Figure 4). Thus, the stepwise approach identified 15 true positive BED cases and 616 true negative BED cases, increasing the specificity to 94% compared with 87% for DEPS‐10 alone and 67% for PAID items alone. Of the participants who reached both cut‐off scores (PAID and DEPS‐10), 27.8% were found to have a BED at the time of the interview (PPV).

### 3.4 Binomial logistic regression analysis

The DEPS‐10 was significantly associated with a higher risk of having a clinical diagnosis of BED, while controlling for demographics, HbA1c, diabetes type and diabetes complications, as well as the *z*‐standardised scores of the food‐related items of the PAID (Table S9). Before entering DEPS‐10 into the model, the PAID items were found to have a significant contribution to the prediction of BED (*ß* = 0.84 ± 0.22, *p* < 0.001, OR = 2.3, 95% CI: 1.5; 3.6). After entering DEPS‐10 into the regression model, the PAID items remained significant, but the significance was reduced (*ß* = 0.53 ± 0.25, *p* = 0.04, OR = 1.7, 95% CI: 1.0; 2.8). The *z*‐standardised DEPS‐10 sum score showed an estimated odds ratio of 2.5 (95% CI: 1.7; 3.8), indicating that with each increase of one standard deviation in the sum score of the DEPS‐10, the risk of having a BED increased by 2.5 times.

## 4 DISCUSSION

The BED point prevalence among participants with type 2 diabetes was 4.3%, in line with previous literature.^3^, ^4^, ^5^, ^6^, ^7^ However, 2.9% of participants with type 1 diabetes met the BED criteria, which is a lower prevalence than in previous studies,^8^, ^9^ possibly due to the higher age of the present sample. Given BED prevalence rates, high specificity in screening tools is crucial to avoid unsettling patients with false positive results.

The ROC analysis showed the clinical usefulness of the DEPS‐10,^29^ with a 92% AUC indicating strong discriminatory power. The DEPS‐10 exceeds the AUC of the PAID food items by 10%. The binomial logistic regression analysis supports the results of the ROC analysis, showing a significant contribution of the DEPS‐10 to the prediction of BED even when controlling for diabetes‐specific variables.

Within the ROC analysis, an optimal cut‐off value of ≥15 for the DEPS‐10 was identified with a high specificity and sensitivity for the BED point prevalence. Compared with the DEPS‐R in the sample of participants with rapid‐acting insulin treatment, the DEPS‐10 showed a better screening performance than the DEPS‐R for BED point prevalence, possibly due to the items of the DEPS‐R addressing insulin‐specific compensatory behaviours not relevant for BED.

The rather low PPV of the DEPS‐10 (19.6%) is comparable to the screening instrument BES with a PPV of 26% in previous literature.^30^ Moreover, the low PPV of the DEPS‐10 may also be due to the wider scope of the diabetes‐specific screening instrument, including other disordered eating behaviours.

To enhance specificity and feasibility in routine care, a stepwise approach using the PAID as a primary screening tool for diabetes distress,^23^ followed by the DEPS‐10, was evaluated. Using a cut‐off of ≥6 for the PAID food items followed by the DEPS‐10 increased the specificity. The stepwise approach raised the PPV to 28%, reducing the number of interviews needed to identify one BED case from seven (single‐use PAID) or five (single‐use DEPS‐10) to four. Healthcare professionals are required to react with further diagnostics in case of positive screening. Doing so would have led to 94 negative diagnostic interviews after the PAID screening, costing significant time in clinical care and risking patient well‐being with false positive results. The second screening via DEPS‐10 reduced the number of false positive BED cases to 39 participants. Notably, persons who were false‐positively screened for BED—meeting the DEPS‐10 cut‐off, but not full BED criteria—may still experience clinically relevant binge episodes or other disordered eating behaviours, suggesting further consideration for tailored support or behavioural intervention. The stepwise approach may build on existing routines with common PAID screening in clinical practice, saving valuable time for health professionals. Yet, the first screening step via PAID overlooked eight participants meeting full BED criteria due to limited sensitivity of the PAID items. For more sensitive screening in populations at risk, we recommend direct screening via DEPS‐10.

Participants meeting the DEPS‐10 cut‐off showed negative clinical outcomes which are also linked to both BED^1^ and disordered eating in a broader sense in recent literature.^13^, ^31^ Participants with DEPS‐10 ≥ 15 had significantly more diabetes distress, more depressive and anxiety symptoms, and higher BMI and HbA1c than participants with scores below the cut‐off. These associations have also been reported for disordered eating behaviours, including compensatory behaviours.^13^, ^31^ Notably, the effect sizes for the differences in clinical outcomes were higher when comparing the groups above and below the DEPS‐10 cut‐off than when distinguishing between participants with and without BED. This finding supports the utility and clinical relevance of the DEPS‐10 not only for BED but also for disordered eating behaviours exceeding the scope of BED diagnosis, which are also associated with a mental health burden.^1^, ^12^

The DEPS‐10 allows for time‐efficient yet broad screening of disordered eating in routine diabetes care, independent of treatment regimen and with more focus on BE compared with the original DEPS‐R. The DEPS‐10 is shorter than many BE scales and the diabetes‐specific items may help to overcome under‐ and overestimation of BE in generic scales.^13^ The DEPS‐10 and the stepwise approach can help overcome challenges in the detection of BED, including limited time and knowledge in routine clinical care.^4^, ^6^, ^14^ If not detected, BED cannot be treated effectively,^4^ leading to unfavourable glycaemic and psychological outcomes for people with diabetes.^1^, ^11^, ^13^ The stepwise approach enhances existing procedures, like the PAID diabetes distress screening, by adding the DEPS‐10 as a second time‐efficient step that is applicable across all treatment regimens. Barriers experienced and internalised by people with BE when encountering healthcare professionals, including shame and fear of stigmatisation^4^, ^32^, may be addressed by offering a new way to introduce the topic of eating behaviour via the DEPS‐10.

### 4.1 Limitations and strengths

In one fifth of the sample, higher DEPS‐10 scores may have been influenced by GLP‐1 therapy, or they may reflect inherent differences between diabetes types. Furthermore, the PPV of the DEPS‐10 was rather low, possibly due to the broader scope of disordered eating behaviour of the DEPS‐10. Because of the present study's focus on BED, the extent of the DEPS‐10 addressing compensatory behaviours and other eating disorders, such as anorexia or bulimia nervosa, was not evaluated in this study. In populations with a higher risk for insulin purging behaviour, i.e. young women with type 1 diabetes, we highly recommend using the DEPS‐R as a valid instrument for disordered eating, including insulin‐specific compensatory behaviours.

To the author's knowledge, this study is the first to introduce a non‐insulin‐specific version of the DEPS‐R and test its performance in the detection of BED assessed via clinical interviews in a large sample of people with diabetes. Few studies have tested the clinical performance of the DEPS‐R via the gold standard of clinical interviews^21^, ^22^ in significantly smaller samples.

## 5 CONCLUSIONS

In summary, the study highlights the need for diabetes‐specific BED screening and presents the DEPS‐10 as a valid and efficient tool for detection of BED and disordered eating behaviours in clinical practice. A two‐step approach using the PAID followed by the DEPS‐10 can be a feasible and time‐efficient procedure in routine care. Future research should evaluate the DEPS‐10's screening performance for other eating disorders, e.g., anorexia and bulimia nervosa, in individuals with insulin‐independent diabetes.

## AUTHOR CONTRIBUTIONS

Laura Klinker designed the study, collected the data, analysed the data and drafted the manuscript. Andreas Schmitt designed the study, collected the data, discussed the findings and revised the manuscript. Dominic Ehrmann, Norbert Hermanns and Bernhard Kulzer designed the study, discussed the findings and revised the manuscript.

## FUNDING INFORMATION

This study was funded by the German Center for Diabetes Research (DZD) (grant number: 82DZD1102A). The funder was not involved in decisions regarding study design; collection, analysis and interpretation of the data; writing of the report; and its submission for publication.

## CONFLICT OF INTEREST STATEMENT

All authors declare that they have no conflicts of interest related to this article.

## Supporting information


Data S1.
