# Supplementary material for: Detecting clinical cases of binge eating in diabetes care: Introducing the Diabetes Eating Problem Survey‐10 (DEPS‐10) for type 1 and type 2 diabetes
Source: Diabet Med. 2025 May 29;42(8):e70060. doi: 10.1111/dme.70060 (PMC12257434; doi:10.1111/dme.70060)

**Supplementary Information**

1. Assessing disordered eating
   1. S1: DSM-5 criteria for binge eating disorder
   2. S2: Introducing non-insulin DEPS-10: Revision of DEPS-R
2. Binge eating disorder (BED) in the sample
   1. S3: BED vs. No BED in 12-month prevalence: Glycaemic and mental health outcomes
3. Psychometric properties of DEPS-10
   1. S4: Mean scores of DEPS-10
   2. S5: Exploratory factor analysis: Two-factor solution
   3. S6: Inter-Item-Correlations of DEPS-10
   4. S7: Pearson Correlations: DEPS-10 and associated outcomes
   5. S8: DEPS-10≤15 vs. DEPS-10≥15: Glycaemic and mental health outcomes
   6. SF1: ROC-Curves: DEPS-10 and DEPS-R: Participants with and without rapid-acting insulin treatment
   7. S9: Binomial regression analysis
4. Sensitivity analysis when excluding participants with type 3 diabetes
   1. SF2: ROC-Curves of the DEPS-10 and food-related items of the PAID in predicting the point prevalence of BED when excluding participants with type 3 diabetes
5. **Assessing disordered eating**

***1a. DSM-5 criteria for binge eating disorder***

*Supplementary Table S1*

|  | DSM-5 criteria for a binge eating episode:  *(at least 3 of the 5 listed criteria are met)* | DSM-5 criteria for binge eating disorder (BED) |
| --- | --- | --- |
| 1 | Eating faster than normal | - At least one binge eating episode per week for 3 months - Binge eating episodes are not followed by inappropriate compensatory behaviour such as purging, use of laxantia or insulin-purging |
| 2 | Eating until feeling uncomfortably full |  |
| 3 | Eating large amounts without the sense of physical hunger |  |
| 4 | Eating alone because of shame/embarrassment |  |
| 5 | Feelings of disgust, guilt or depression after overeating |  |

***1b.*** ***Introducing non-insulin DEPS-10: Revision of DEPS-R***

|  | DEPS-R | Insulin purging behaviour | Insulin purging attitudes | |  |
| --- | --- | --- | --- | --- | --- |
| 1 | Losing weight is an important goal to me |  | |  | |
| 2 | I skip meals and/or snacks |  | |  | |
| 3 | Other people have told me that my eating is out of control |  | |  | |
| 4 | When I overeat, I don't take enough insulin to cover the food | x | |  | |
| 5 | I eat more when I am alone than when I am with others |  | |  | |
| 6 | I feel that it's difficult to lose weight and control my diabetes at the same time |  | | x | |
| 7 | I avoid checking my blood sugar when I feel like it is out of range |  | |  | |
| 8 | I make myself vomit |  | |  | |
| 9 | I try to keep my blood sugar high so that I will lose weight |  | |  | |
| 10 | I eat in a way to get ketones | x | |  | |
| 11 | I feel fat when I take all my insulin |  | | x | |
| 12 | Other people tell me to take better care of my diabetes |  | |  | |
| 13 | After I overeat, I skip my next insulin dose | x | |  | |
| 14 | I feel that my eating is out of control |  | |  | |
| 15 | I alternate between eating very little and eating huge amounts |  | |  | |
| 16 | I would rather be thin that to have good control of my diabetes |  | | x | |

*Supplementary Table S2*

*Note.* All marked items were excluded in the DEPS-10.

1. ***Binge eating disorder (BED) in the sample***

***2a. BED vs. No BED in 12-month prevalence: Glycaemic and mental health outcomes***

*Supplementary Table S3*

|  | No BED in 12-month-prevalence  (n=649) | BED in 12-month-prevalence  (n=30) | p | d |
| --- | --- | --- | --- | --- |
| HbA1c (mmol)  (%)  BMI | 58±15 (7.4±1.4)  29.2±5.9 | 62±18  (7.8±1.7)  34.8±6.8 ** | *.127*  *<.001* | 0.1  0.4 |
| Mental health PAID  PHQ-9  GAD-7 | 24.4±18.2  6.3±4.8 5.8±4.4 | 39.5±19.6 ** 12.6±6.8 ** 9.9±6.0 ** | *<.001 <.001 <.001* | 0.3  0.4  0.3 |
| Comorbidities  Any depressive episode^a^  Diabetes complications^b^ | 67(10.3%)  334 (51.9%) | 11(36.7%) **  18 (60.0%) | *<.001*  *.383* | 0.3  0.07 |

*Note.* BED, binge eating disorder; BMI, body-mass-index; PAID, Problem Areas in Diabetes; PHQ-9, Patient Health Questionnaire-9; GAD-7, General Anxiety Disorder-7. Mann-Whitney U-tests were conducted for all interval-scaled variables and χ²-tests for dichotomic variables (depressive episode and diabetes complications).
^a^including any form of depression or dysthymia
^b^including retinopathy, nephropathy, polyneuropathy and diabetic foot syndrome

1. **Psychometric properties of the DEPS-10**

***3a. Mean scores of DEPS-10***

*Supplementary Table S4*

| Nr. | Item | *M* | *SD* |
| --- | --- | --- | --- |
| 1 | Losing weight is an important goal to me | .94 | 1.61 |
| 2 | I skip meals and/or snacks | .60 | 1.15 |
| 3 | Other people have told me that my eating is out of control | .15 | .57 |
| 5 | I eat more when I am alone than when I am with others | .33 | .88 |
| 7 | I avoid checking my blood sugar when I feel like it is out of range | .27 | .83 |
| 8 | I make myself vomit | .01 | .09 |
| 9 | I try to keep my blood sugar high so that I will lose weight | .02 | .25 |
| 12 | Other people tell me to take better care of my diabetes | .16 | .58 |
| 14 | I feel that my eating is out of control | .27 | .79 |
| 15 | I alternate between eating very little and eating huge amounts | .34 | .83 |

*Note.* M, Mean; SD, Standard deviation.

***3b. Exploratory factor analysis: Two-factor solution***

*Supplementary Table S5*

|  | | | **Patternmatrix** | | |  |
| --- | --- | --- | --- | --- | --- | --- |
|  | | Items | | | Factor | |
| Nr. | |  |  |  | 1 | 2 |
| 14 | | I feel that my eating is out of control | | .870 |  |  |
| 3 | | Other people have told me that my eating is out of control | | .749 |  |  |
| 12 | | Other people tell me to take better care of my diabetes | | .711 |  |  |
| 15 | | I alternate between eating very little and eating huge amounts | | .646 | -.217 |  |
| 5 | | I eat more when I am alone than when I am with others | | .590 | -.256 |  |
| 7 | | I avoid checking my blood sugar when I feel like it is out of range | | .479 |  |  |
| 1 | | Losing weight is an important goal to me | |  | -.963 |  |
| 2 | | I skip meals and/or snacks | |  | -.776 |  |
| 8 | | I make myself vomit | |  |  |  |
| 9 | | I try to keep my blood sugar high so that I will lose weight | |  |  |  |

*Note*. Extraction method: Principal axis factor analysis. Rotation method: Oblimin with Kaiser normalisation. The rotation has converged in 5 iterations.

The exploratory factor analysis with oblimin rotation suggests a two-factor-solution with eigenvalues > 1 explaining 57,8% of the variance.

The KMO value of the two-factor solution was 0.87, and the Bartlett test of sphericity reached statistical significance (χ² = 3067.8, df =45, p < .001). The first factor comprises six items addressing binge eating/loss of control, the second factor consists of two items depicting dieting behaviour, the items 08 and 09 on compensatory behaviour load on neither of the two factors.

**3c. *Inter-Item-Correlations of DEPS-10***

*Supplementary Table S6*

|  | **1** | **2** | **3** | **5** | **7** | **8** | **9** | **12** | **14** | **15** |
| --- | --- | --- | --- | --- | --- | --- | --- | --- | --- | --- |
| **1** | 1.00 |  |  |  |  |  |  |  |  |  |
| **2** | .79 | 1.00 | . |  |  |  |  |  |  |  |
| **3** | .38 | .40 | 1.00 |  |  |  |  |  |  |  |
| **5** | .57 | .53 | .47 | 1.00 |  |  |  |  |  |  |
| **7** | .44 | .45 | .36 | .53 | 1.00 |  |  |  |  |  |
| **8** | .10 | .10 | .07 | .21 | .09 | 1.00 |  |  |  |  |
| **9** | .19 | .13 | .03 | .18 | .05 | -.01 | 1.00 |  |  |  |
| **12** | .37 | .38 | .53 | .45 | .45 | .14 | .02 | 1.00 |  |  |
| **14** | .51 | .45 | .63 | .67 | .50 | .18 | .20 | .54 | 1.00 |  |
| **15** | .59 | .54 | .54 | .66 | .48 | .16 | .12 | .50 | .68 | 1.00 |

*Note.* The item numbering is based on the original scale DEPS-16 to ensure comparability.

**3d. *Pearson Correlations: DEPS-10 and associated outcomes***

*Supplementary Table S7*

|  | DEPS-10 | *p* |
| --- | --- | --- |
| PAID sum score | .427 | <.001 |
| Food-related problems (PAID) | .472 | <.001 |
| BMI | .321 | <.001 |
| HbA1c | .254 | <.001 |

*Note.* PAID, Problem Areas In Diabetes Scale; BMI, body-mass-index; HbA1c, haemoglobin A1c.

Highly significant correlations between the DEPS-10 and PAID as well as BMI and HbA1c indicate good construct validity of the scale.

***3e. DEPS-10≤15 vs. DEPS-10≥15: Glycaemic and mental health outcomes***

*Supplementary Table S8*

|  | DEPS-10≤15 (n=572) | DEPS-10≥15  (n=107) | p | d |
| --- | --- | --- | --- | --- |
| BMI | 28.9±5.8 | 32.2±6.9 | *<.001* | 0.4 |
| Mental health PAID  PHQ-9  GAD-7 | 22.2±17.2  5.8±4.6  5.4±4.3 | 40.3*±*18.1  11.0±5.4  9.2±5.0 | *<.001 <.001 <.001* | 0.7  0.8  0.6 |
| Comorbidities  Any depressive episode^a^  Diabetes complications ^b^ | 60 (10%)  299 (52.7%) | 24 (22%)  53 (49.5%) | *<.001*  *.543* | 0.3  0.05 |
| Glycaemic outcomes  HbA1c (mmol)  (%) | 56±14  (7.3±1.3) | 66±22  (8.1±1.8) | *<.001* | 0.4 |
| Subsample (n=350)  Time-in-range (%)  Time above 180mg/d (%)  Glucose CV (%) | *(n=304)*  69.8±16.3 27.4±16.5  33.4±7.8 | *(n=46)*  65.0±21.1  33.3±20.7  31.9±9.0 | *.106*  *< .05*  *.506* | .2  .2  .07 |

*Note.* BMI, body-mass-index; PAID, Problem Areas in Diabetes; PHQ-9, Patient Health Questionnaire-9; GAD-7, General Anxiety Disorder-7; d, Cohen’s d. Mann-Whitney U-tests were conducted for all interval-scaled variables and χ²-tests for dichotomic variables (depressive episode and diabetes complications). ^a^including any form of depression or dysthymia
^b^including retinopathy, nephropathy, polyneuropathy and diabetic foot syndrome

***3f. ROC-Curves: DEPS-10 and DEPS-R: Participants with and without rapid-acting insulin treatment***

*Supplementary Figure SF1*


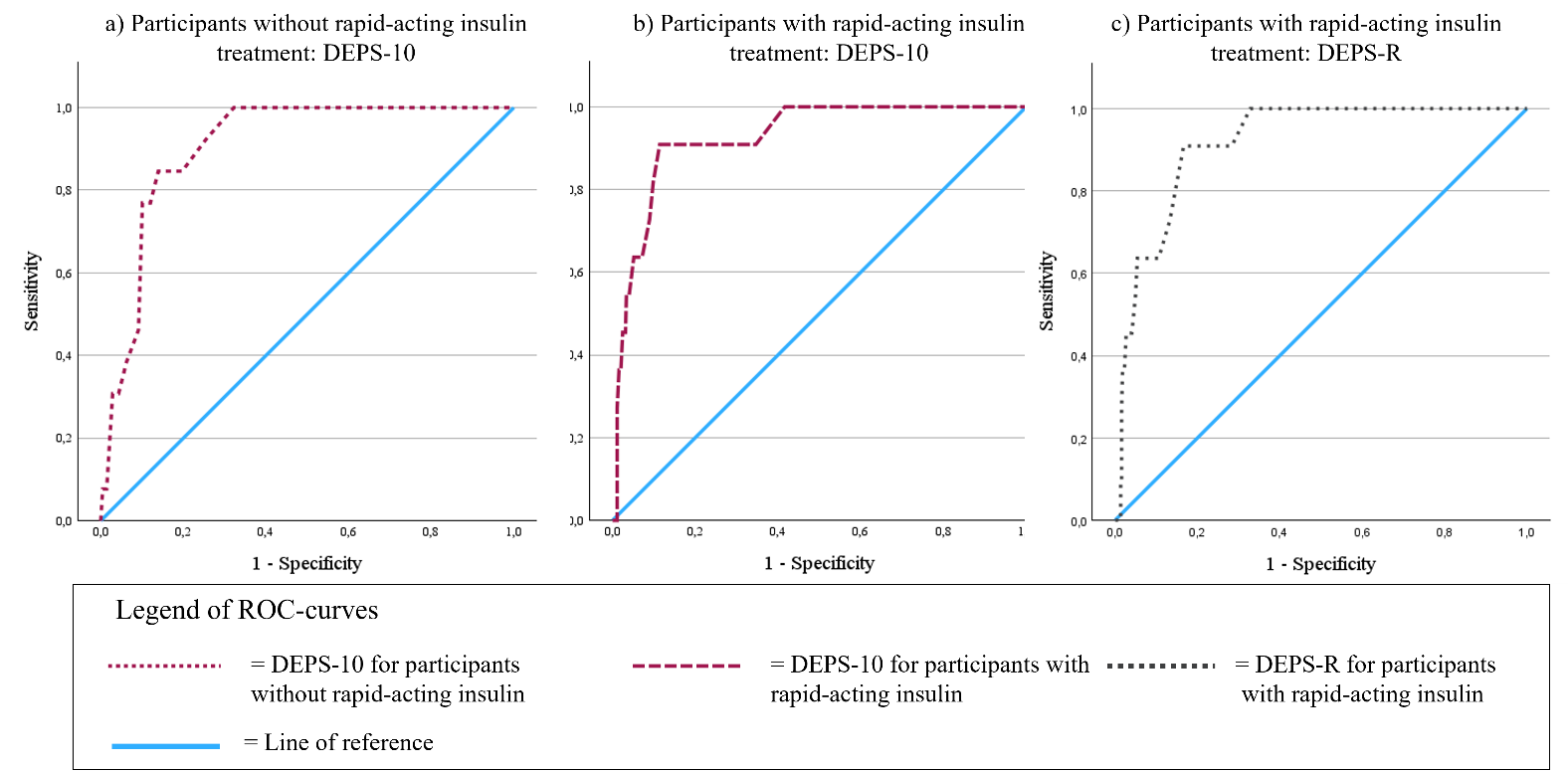


***3g.* Binomial logistic regression with point prevalence of BED as criterion**

*Supplementary Table S9*

| *Pre-dictors* | | *ß* | | *SE* | *P* | *Odds Ratio (95% CI)* | *ß* | *SE* | *P* | *Odds Ratio (95% CI)* | *ß* | *SE* | *P* | *Odds Ratio (95% CI)* |  |
| --- | --- | --- | --- | --- | --- | --- | --- | --- | --- | --- | --- | --- | --- | --- | --- |
| *First Block: Demographics and diabetes-related characteristics* | | | | | | | *Second Block: food-related problems (PAID)* | | | | *Third Block: DEPS-10* | | | | |
| Sex | | .02 | | .44 | *.70* | 1.19  (.50;2.81) | -.16 | .47 | *.74* | .86  (.34;2.13) | -.20 | .50 | *.70* | .82  (.31;2.17) |  |
| Age | **-.05** | | | **.02** | ***.001*** | **.95  (.93;.98)** | **-.03** | **.02** | ***.03*** | **.97 (.94;1.00)** | -.01 | .02 | *.55* | .99  (.96;1.02) |  |
| BMI | | **.08** | | **.03** | ***.004*** | **1.09 (1.03;1.15)** | **.07** | **.03** | ***.03*** | **1.07 (1.01;1.14)** | .03 | .03 | *.38* | 1.03  (.96;1.10) |  |
| Type of diabetes | | **.62** | | **.28** | ***.03*** | **1.86  (1.08-3.20)** | .28 | .30 | *.35* | 1.32  (.73-2.39) | .22 | .33 | *.51* | 1.25  (.65-2.40) |  |
| HbA1c | | **.02** | | **.01** | ***.07*** | **1.02 (1.00;1.04)** | .01 | .01 | *.25* | 1.01 (.99;1.04) | .004 | .01 | *.74* | 1.00  (.98;1.03) |  |
| DM compli-cations | | .20 | | .24 | *.41* | 1.22  (.77; 1.93) | .14 | .22 | *.58* | 1.14  (.71; 1.85) | .16 | .27 | *.57* | 1.17  (.69; 1.98) |  |
| Food-related problems* (PAID) | | | | | | | **.84** | **.22** | ***<.001*** | **2.31  (1.49; 3.58)** | **.53** | **.25** | ***.04*** | **1.70  (1.04; 2.77)** |  |
| DEPS-10* | | |  |  |  |  |  |  |  |  | **.93** | **.20** | ***< .001*** | **2.53  (1.70; 3.78)** |  |

*Note.* BED, binge eating disorder; BMI, body mass index; HbA1c, glycated haemoglobin in mmol/mol; DM, diabetes mellitus; PAID, Problem Areas in Diabetes Scale; DEPS-10, Diabetes Eating Problem Survey-10.
*Data of the scales have been z-standardised.

1. **Sensitivity analysis when excluding participants with type 3 diabetes**

**4a. ROC-Curves of the DEPS-10 and food-related items of the PAID in predicting the point prevalence of BED when excluding participants with type 3 diabetes**

*Supplementary Figure SF2*


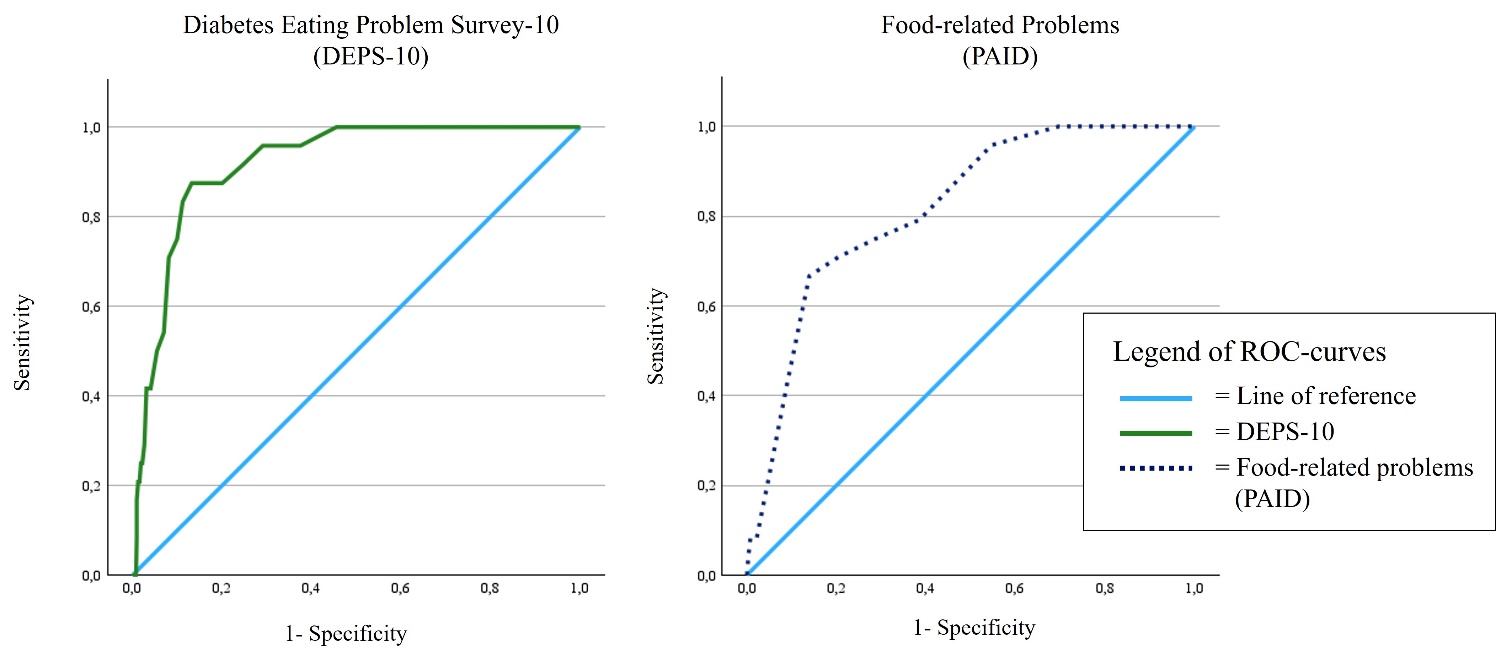

Supplement: Supplementary file 1 — Data S1. [file DME-42-e70060-s001.docx]
